# A non-linear regression method for estimation of gene–environment heritability

**DOI:** 10.1093/bioinformatics/btaa1079

**Published:** 2020-12-26

**Authors:** Matthew Kerin, Jonathan Marchini

**Affiliations:** Wellcome Trust Center for Human Genetics, Oxford, OX3 7BN, UK; Regeneron Genetics Center, Tarrytown, NY 10591, USA

## Abstract

**Motivation:**

Gene–environment (GxE) interactions are one of the least studied aspects of the genetic architecture of human traits and diseases. The environment of an individual is inherently high dimensional, evolves through time and can be expensive and time consuming to measure. The UK Biobank study, with all 500 000 participants having undergone an extensive baseline questionnaire, represents a unique opportunity to assess GxE heritability for many traits and diseases in a well powered setting.

**Results:**

We have developed a randomized Haseman–Elston non-linear regression method applicable when many environmental variables have been measured on each individual. The method (GPLEMMA) simultaneously estimates a linear environmental score (ES) and its GxE heritability. We compare the method via simulation to a whole-genome regression approach (LEMMA) for estimating GxE heritability. We show that GPLEMMA is more computationally efficient than LEMMA on large datasets, and produces results highly correlated with those from LEMMA when applied to simulated data and real data from the UK Biobank.

**Availability and implementation:**

Software implementing the GPLEMMA method is available from https://jmarchini.org/gplemma/.

**Supplementary information:**

Supplementary data are available at *Bioinformatics* online.

## 1 Introduction

The advent of genome-wide association studies (The Wellcome Trust Case Control Consortium, 2007) has catalyzed a huge number of discoveries linking genetic markers to many human complex diseases and traits. For the most part, these discoveries have involved common variants that confer relatively small amounts of risk and only account for a small proportion of the phenotypic variance of a trait (Manolio *et al.*, 2009). This has led to a surge of interest in methods and applications that measure the joint contribution to phenotypic variance of all measured variants throughout the genome (SNP heritability), and in testing individual variants within this framework. Most notably the seminal paper of Yang et al. (2010), who used a linear mixed model (LMM) to show that the majority of missing heritability for height could be explained by genetic variation by common SNPs (Yang *et al.*, 2010). When testing variants for association these LMMs can reduce false positive associations due to population structure, and improve power by implicitly conditioning on other loci across the genome (Listgarten *et al.*, 2012; Loh *et al.*, 2015; Yang *et al.*, 2014). These methods model the unobserved polygenic contribution as a multivariate Gaussian with covariance structure proportional to a genetic relationship matrix (GRM) (Eskin *et al.*, 2008; Lippert *et al.*, 2011; Zhou and Stephens, 2012). This approach is mathematically equivalent to a whole genome regression (WGR) model with a Gaussian prior over SNP effects (Listgarten *et al.*, 2012).

Subsequent research has shown that the simplest LMMs make assumptions about the relationship between minor allele frequency (MAF), linkage disequilibrium (LD) and trait architecture that may not hold up in practice (Evans *et al.*, 2018; Speed *et al.*, 2012) and generalizations have been proposed that stratify variance into different components by MAF and LD (Speed *et al.*, 2012, 2017; Yang *et al.*, 2015). Other flexible approaches have been proposed in both the animal breeding (de los Campos *et al.*, 2013; Hayes *et al.*, 2001) and human literature (Carbonetto and Stephens, 2012; Logsdon *et al.*, 2010; Zhou *et al.*, 2013) to allow different prior distributions that better capture SNPs of small and large effects. For example, a mixture of Gaussians (MoG) prior can increase power to detect associated loci in some (but not all) complex traits (Carbonetto and Stephens, 2012; Loh *et al.*, 2015). Other methods have been proposed that estimate heritability only from summary statistics and LD reference panels (Bulik-Sullivan *et al.*, 2015; Speed and Balding, 2019). Heritability can also be estimated using Haseman–Elston regression (Haseman and Elston, 1972) and has recently been extended using a randomized approach (Wu and Sankararaman, 2018) that has O(NM) computational complexity and works for multiple variance components (Pazokitoroudi *et al.*, 2020). Other recent work has shown that LMM approaches such as these are not able to disentangle direct and indirect genetic effects, the balance of which will vary depending on the trait being studied (Young *et al.*, 2018).

There has been less exploration of methods for estimating heritability that account for gene–environment interactions. One interesting approach has proposed using spatial location as a surrogate for environment (Heckerman *et al.*, 2016) using a three component LMM—one based on genomic variants, one based on measured spatial location as a proxy for environmental effects, and a gene–environment component, modelled as the Hadamard product of the genomic and spatial covariance matrices. Other authors have used this method to account for gene-gene interactions (Crawford *et al.*, 2017; Ober *et al.*, 2015).

Modelling gene–environment interactions when many different environmental variables are measured is a more challenging problem. If several environmental variables drive interactions at individual loci, or if an unobserved environment that drives interactions is better reflected by a combination of observed environments, it can make sense to include all variables in a joint model. StructLMM (Moore *et al.*, 2019) focuses on detecting GxE interactions at individual markers. Environmental similarity between individuals is modelled (over multiple environments) as a random effect, and each SNP is tested independently for GxE interactions. However, this approach does not model the genome wide contribution of all the markers, which is often a major component of phenotypic variance.

We recently proposed a WGR approach called LEMMA applicable to large human datasets such as UK Biobank, where many potential environmental variables are available (Kerin and Marchini, 2020). The LEMMA regression model includes main effects of each genotyped SNP across the genome, and also interactions of each SNP with a environmental score (ES), that is a linear combination of the environmental variables. The ES is estimated as part of the method using a Variational Bayes algorithm to fit the WGR model. The model uses mixture of Gaussian (MoG) priors on main and GxE SNP effects, that allow for a range of different genetic architectures from polygenic to sparse genetic effects (Carbonetto and Stephens, 2012; Logsdon *et al.*, 2010; Zhou *et al.*, 2013). The ES can be readily interpreted and its main use is to test for GxE interactions one variant at a time, typically at a larger set of imputed SNPs in the dataset. However, the ES can also be used to estimate the proportion of phenotypic variability that is explained by GxE interactions (SNP GxE heritability), using a two component randomized Haseman–Elston (RHE) regression (Pazokitoroudi *et al.*, 2020).

The main contribution of this article is to combine the estimation of the LEMMA ES into a stand-alone RHE framework. This results in a non-linear optimization problem that we solve using the Levenburg-Marquardt (LM) algorithm. The method implicitly assumes a Gaussian prior on main effect and GxE effect sizes. We also propose a separate RHE method that estimates the independent GxE contribution of each measured environmental variable. We set out the differences between these two models and present a simulation study to compare them to LEMMA. We show that GPLEMMA is more computationally efficient than LEMMA on large datasets. We also apply the method to UK Biobank data and show that GPLEMMA produces estimates very close to LEMMA. Software implementing the GPLEMMA algorithm in C++ is available at https://jmarchini.org/gplemma/.

## 2 Materials and methods

### 2.1 Modelling SNP heritability

The simplest model for estimating SNP heritability has the form

$$y=X\beta +e,\;\beta_{l}\sim N\left(0,\frac{\sigma_{g}^{2}}{M}\right),\;e\sim N\left(0,\sigma_{e}^{2}\right)$$

where *y* is a continuous phenotype, *X* is an *N *×* M* matrix of genotypes that has been normalized to have column mean zero and column variance one, and *β* is an *M*-vector of SNP effect sizes. Integrating out *β* leads to the variance component model

$$y\sim N\left(0,\sigma_{g}^{2}K+\sigma_{e}^{2}I\right),$$

where $K=\frac{XX^{T}}{M}$ is known as the genomic relationship matrix (GRM) (Yang *et al.*, 2010). Estimating the two parameters in this model *σ_g_* and *σ_e_* leads to an estimate of SNP heritability of $h^{2}=\frac{\sigma_{g}^{2}}{\sigma_{g}^{2}+\sigma_{e}^{2}}$. This is commonly referred to in the literature as the single component model. Subsequent research has shown that the single component model makes assumptions about the relationship between minor allele frequency (MAF), linkage disequilibrium (LD) and trait architecture that may not hold up in practice (Evans *et al.*, 2018; Speed *et al.*, 2012). There have been many follow up methods, including; generalizations that stratify variance into different components by MAF and LD (Yang *et al.*, 2015), approaches that assign different weights for the GRM (Speed *et al.*, 2012, 2017), methods that replace the Gaussian prior on *β* with a spike and slab on SNP effect sizes (Powell *et al.*, 2018) and methods that estimate heritability only from summary statistics and LD reference panels (Bulik-Sullivan *et al.*, 2015; Finucane *et al.*, 2015).

### 2.2 Randomized Haseman–Elston regression

An alternative method used to compute heritability is known as Haseman–Elston (HE) regression (Haseman and Elston, 1972). HE-regression is a method of moments (MoM) estimator that optimizes variance components $\left(\sigma_{g}^{2},\sigma_{e}^{2}\right)$ in order to minimize the squared difference between the observed and expected trait covariances. The MoM estimator $\left({\hat{\sigma }}_{g}^{2},{\hat{\sigma }}_{e}^{2}\right)$ can be obtained by solving the minimization

$$\arg\;\min_{\sigma_{g}^{2},\sigma_{e}^{2}}||yy^{T}-(\sigma_{g}^{2}K+\sigma_{e}^{2}I)|{|}_{F}^{2}$$

or equivalently by solving the linear regression problem

$$\text{vec}\left(yy^{T}\right)=\sigma_{g}^{2}\text{vec}\left(K\right)+\sigma_{e}^{2}\text{vec}\left(I\right)+\epsilon \prime$$

where vec(A) is the vectorization operator that transforms an *N *×* M* matrix into an *NM*-vector. In matrix format, both of these forms correspond to solving the following linear system

(1) $$\left(\begin{array}{cc} \text{tr}\left(K^{2}\right) & \text{tr}\left(K\right)\\ \text{tr}\left(K\right) & N \end{array}\right)\left(\begin{array}{c} \sigma_{\beta }^{2}\\ \sigma_{e}^{2} \end{array}\right)=\left(\begin{array}{c} y^{T}Ky\\ y^{T}y \end{array}\right)$$

HE-regression methods are widely acknowledged to be more computationally efficient (Golan *et al.*, 2014; Wu and Sankararaman, 2018; Yang *et al.*, 2017) and do not require any assumptions on the phenotype distribution beyond the covariance structure (Golan *et al.*, 2014) (in contrast to maximum-likelihood estimators). However, HE-regression based estimates typically have higher variance (Yang *et al.*, 2017), thus implying that they have less power.

Recent method developments (Pazokitoroudi *et al.*, 2020; Wu and Sankararaman, 2018) have shown that a randomized HE-regression (RHE) approach can be used to compute efficiently on genetic datasets with hundreds of thousands of samples. Wu and Sankararaman (2018) observed that Equation (1) can be solved efficiently without ever having to explicitly compute the kinship matrix *K* by using Hutchinson’s estimator (Hutchinson, 1990), which states that $\text{tr}\left(A\right)=E\left[z^{T}Az\right]$ for any matrix where *z* is a random vector with mean zero and covariance given by the identity matrix. The proposed method involves approximating tr(K) and $\text{tr}\left(K^{2}\right)$ using only matrix vector multiplications with the genotype matrix *X*, to compute the following expressions

$$\begin{array}{c} \text{tr}\left(K\right)\approx \frac{1}{B}\frac{1}{M^{2}}\sum_{b}||X^{T}z_{b}|{|}_{2}^{2},\\ \text{tr}\left(K^{2}\right)\approx \frac{1}{B}\frac{1}{M^{2}}\sum_{b}||XX^{T}z_{b}|{|}_{2}^{2}. \end{array}$$

Thus an approximate solution can be obtained in O(NMB) time, where *B* denotes a relatively small number of random samples. Subsequent work by Pazokitoroudi *et al.* extended this approach to a multiple component model (Pazokitoroudi *et al.*, 2020)

$$y\sim N\left(0,\sum_{k}\sigma_{k}^{2}K_{k}+I\sigma_{e}^{2}\right).$$

With parameter estimates obtained as solution to the linear system given by

(2) $$\left(\begin{array}{cc} T & b\\ b^{T} & N \end{array}\right)\left(\begin{array}{c} \sigma_{\beta }^{2}\\ \sigma_{e}^{2} \end{array}\right)=\left(\begin{array}{c} c\\ N \end{array}\right),$$

where $T_{kl}=\text{tr}\left(K_{k}K_{l}\right),\;b_{k}=\text{tr}\left(K_{k}\right)$ and $c_{k}=y^{T}K_{k}y$. Finally, both papers show how to efficiently control for covariates by projecting them out of all terms in the system of equations. Thus with covariates included the multiple component model becomes

$$y\sim N\left(C\alpha ,\sum_{k}\sigma_{k}^{2}K_{k}+I\sigma_{e}^{2}\right),$$

and terms in the subsequent linear system are given by $T_{kl}=\text{tr}\left(WK_{k}WK_{l}W\right),\;b_{k}=\text{tr}\left(WK_{k}W\right)$ and $c_{k}=y^{T}WK_{k}Wy$, where $W=I_{N}-C^{T}{\left(C^{T}C\right)}^{-1}C$. The GPLEMMA and MEMMA approaches developed in this article use this method of handling covariates.

### 2.3 Modelling GxE heritability

We introduce two extensions of the RHE framework for modelling GxE interactions with multiple environmental variables. In both models we let *E* be an *N *×* L* matrix of environmental variables and *C* be an *N *×* D* matrix of covariates, where both matrixes are normalized such that columns have mean zero and variance one. We use notation *E_l_* to denote the *l*th column of *E*, and diag(x) denotes a null matrix with elements of vector *x* on its diagonal. We note that columns of *E* are always included in *C*, so that *D *>* L*.

#### 2.3.1 MEMMA

The first model assumes that each environmental variable interacts independently with the genome

(3) $$y=C\alpha +X\beta +\sum_{l}\left(E_{l}⊙X\right)\lambda_{l}+\epsilon ,$$

where $\beta \sim N\left(0,\frac{\sigma_{\beta }^{2}}{M}I_{M}\right),\lambda_{l}\sim N\left(0,\frac{\sigma_{w_{l}}^{2}}{M}I_{M}\right),\epsilon \sim N\left(0,\sigma_{e}^{2}I_{N}\right)$ and $E_{l}⊙X$ denotes the element-wise product of *E_l_* with each column of *X*. Integrating out *β* and *λ* leads to the variance component model

$$y\sim N\left(C\alpha ,\sum_{k=1}^{L+2}\theta_{k}K_{k}\right),$$

where $\theta =\left\{\sigma_{\beta }^{2},{\left(\sigma_{w_{l}}^{2}\right)}_{l=1}^{L},\sigma_{e}^{2}\right\},\;F_{k}=E_{k}⊙X$ and

$$K_{k}=\{\begin{array}{cc} \frac{XX^{T}}{M} & \text{if}\;k=1,\\ \frac{F_{k-1}F_{k-1}^{T}}{M} & \text{if}\;1<k\leq L+1,\\ I & \text{if}\;k=L+2. \end{array}$$

Fitting the variance components is done analytically by solving the system of equations Tθ=c where $T_{kl}=\text{tr}\left(WK_{k}WK_{l}W\right),\;c_{k}=y^{T}WK_{k}Wy$ and $W=I_{N}-C{\left(C^{T}C\right)}^{-1}C^{T}$. As shown in the original RHE method (Pazokitoroudi *et al.*, 2020; Wu and Sankararaman, 2018), Hutchinson’s estimator can be used to efficiently estimate *T_kl_*. To do this our software streams SNP markers from a file and computes $y^{T}WXX^{T}Wy$ and the following *N*-vectors

(4) $$u_{b}=XX^{T}Wz_{b},$$

 

(5) $$v_{b,l}=XX^{T}E_{l}Wz_{b},$$

where $z_{b}\sim N(0,I_{N})$ for 1≤b≤B are random *N*-vectors. Then

$$T_{kl}=\frac{1}{M^{2}B}\sum_{b}{\left(\xi_{b}^{k}\right)}^{T}\xi_{b}^{k},$$

where $\xi_{b}^{k}$ is defined as

$$\xi_{b}^{k}=\left\{ \begin{array}{cc} u_{b} & \text{if}\;k=1,\\ v_{b,l} & \text{if}\;1<k\leq L+1,\\ z_{b} & \text{if}\;k=L+2. \end{array}\right.$$

Finally, the variance components are converted to heritability estimates using the following formula

$${\hat{h}}_{k}^{2}=\frac{{\hat{\theta }}_{k}\text{tr}\left(K_{k}\right)}{\sum_{k}{\hat{\theta }}_{k}\text{tr}\left(K_{k}\right)}.$$

We call this approach MEMMA (Multiple Environment Mixed Model Analysis). MEMMA costs O(NMLB) in compute and O(NLB) in RAM.

#### 2.3.2 GPLEMMA

The second model involves the estimating a linear combination of environments, or environmetal score (ES), that interacts with the genome. The model is given by

(6) y=Cα+Xβ+(η⊙X)γ+ϵ,

 

(7) $$\beta \sim N\left(0,\frac{\sigma_{\beta }^{2}}{M}I_{M}\right),$$

 

(8) $$\gamma \sim N\left(0,\frac{\sigma_{\gamma }^{2}}{M}I_{M}\right),$$

where η=Ew is a column vector that we refer to as the linear environmental score (ES). This is the same model used by LEMMA (Kerin and Marchini, 2020), which we include below for completeness, except the mixture of Gaussians priors on SNP effects (*β* and *γ*) have been replaced with Gaussian priors. For this reason, we call this approach GPLEMMA (Gaussian Prior Linear Environment Mixed Model Analysis). Integrating out the SNP effects yields the model

$$y\sim N\left(C\alpha ,\sigma_{\beta }^{2}K+\sigma_{\gamma }^{2}K_{2}(w)+\sigma_{e}^{2}I\right),$$

where $K_{2}(w)=\text{diag}\left(Ew\right)K\text{diag}\left(Ew\right)=\frac{1}{M}\sum_{l,m}w_{l}w_{m}F_{l}F_{m}^{T}$ and $F_{l}=E_{l}⊙X$. Minimizing the squared loss between the expected and observed covariance is equivalent to the following regression problem

(9) $$\text{vec}\left(yy^{T}\right)=\sigma_{\beta }^{2}\text{vec}\left(K\right)+\sum_{l,m}\sigma_{\gamma }^{2}w_{l}w_{m}\text{vec}\left(F_{l}F_{m}^{T}\right)+\sigma_{e}^{2}\text{vec}\left(I\right)+\epsilon \prime .$$

In this format it is clear that optimizing $\sigma_{\beta }^{2},\sigma_{\gamma }^{2},w,\sigma_{e}^{2}$ is a non-linear regression problem. Further, including a parameter for $\sigma_{\gamma }^{2}$ is no longer necessary. From here on we set ${\tilde{w}}_{l}=\sqrt{\sigma_{\gamma }^{2}}w_{l}$ and drop the parameterization without loss of generality.

#### 2.3.3 Levenburg–Marquardt algorithm

We use the Levenburg-Marquardt (LM) algorithm (Zolfaghari *et al.*, 2005), which is commonly used for non-linear least squares problems. The algorithm effectively interpolates between the Gauss-Newton algorithm and the method of steepest gradient descent, by use of an adaptive damping parameter. In this manner, it is more robust than the straight forward Gauss-Newton algorithm but should have faster convergence than a gradient descent approach.

Without loss of generality, consider the model

(10) Y=f(θ)+ϵ,

where f(θ) is a function that is non-linear in the parameters *θ*. Given a starting point *θ*_0_, LM proposes a new point $\theta_{\text{new}}=\theta_{0}+\delta$ by solving the normal equations

(11) $$\left(J{\left(\theta_{0}\right)}^{T}J(\theta_{0})+\mu I\right)\delta =J{\left(\theta_{0}\right)}^{T}\epsilon (\theta_{0}),$$

where $J(\theta_{0})=\frac{\delta f(\theta_{0})}{\delta \theta_{0}}$ and $\epsilon (\theta_{0})=Y-f(\theta_{0})$ are respectively the Jacobian and the residual vector evaluated at *θ*_0_.

If $\theta_{\text{new}}$ has lower squared error than *θ*_0_, then the step is accepted and the adaptive damping parameter *μ* is reduced. Otherwise, *μ* is increased and a new step *δ* is proposed. For small values of *μ*  Equation (11) approximates the quadratic step appropriate for a fully linear problem, whereas for large values of *μ*  Equation (11) behaves more like steepest gradient descent. This allows the algorithm to defensively navigate regions of the parameter space where the model is highly non-linear. If θ+δ reduces the squared error, then the step is accepted and *μ* is reduced, otherwise *μ* is increased and a new step *δ* is proposed.

In summary the LM algorithm requires computation of the matrices $J{\left(\theta \right)}^{T}J(\theta ),\;J{\left(\theta \right)}^{T}\epsilon (\theta )$ at each step, as well as the squared error (which we define as S(θ)). We now give statements of the equations used to compute each of these values, and show that each iteration can be performed in $O(NL^{2}B)$ time.

We apply the LM algorithm with $\theta =\left\{\sigma_{\beta }^{2},w,\sigma_{e}^{2}\right\},\;Y=\text{vec}\left(yy^{T}\right)$ and

$$f(\theta )=\sigma_{\beta }^{2}\text{vec}\left(K\right)+\sum_{l,m}w_{l}w_{m}\text{vec}\left(F_{l}F_{m}^{T}\right)+\sigma_{e}^{2}\text{vec}\left(I\right).$$

Several quantities can be pre-calculated and re-used in the LM algorithm. The *N*-vectors *u_b_*, $v_{b,l}$ and $y^{T}WXX^{T}Wy$ are needed and have been defined above. In addition, GPLEMMA also benefits from the pre-calculation of

$$H_{l,m}=E_{l}^{T}\text{diag}\left(Wy\right)XX^{T}\text{diag}\left(Wy\right)E_{m},\;1\leq l,m\leq L$$

which can also be computed as genotypes are streamed from file.

Let ${\left(J^{T}J\right)}_{\theta_{i},\theta_{j}}$ denote the entry of the *J^TJ^* that corresponds to ${\frac{f(\theta )}{\partial \theta_{i}}}^{T}\frac{f(\theta )}{\partial \theta_{i}}$ for $\theta_{i},\theta_{j}\in \left\{w,\sigma_{\beta }^{2},\sigma_{e}\right\}$ and define the *N*-vector $v_{b}(w)=\sum_{l}w_{l}v_{b,l}$. Then the (L+2)×(L+2) matrix $J{\left(\theta \right)}^{T}J(\theta )$ is given by

$$\begin{array}{c} {\left(J^{T}J\right)}_{w_{l},w_{m}}=\text{tr}\left(\text{diag}\left(\eta \right)K\text{diag}\left(E_{l}\right)\text{diag}\left(E_{m}\right)K\text{diag}\left(\eta \right)\right),\\ =\frac{1}{M^{2}B}\sum_{b}\left(v_{b}{\left(w\right)}^{T}\text{diag}\left(E_{l}\right)\text{diag}\left(E_{m}\right)v_{b}(w)\right),\\ {\left(J^{T}J\right)}_{w_{l},\sigma_{\beta }^{2}}=\text{tr}\left(\text{diag}\left(\eta \right)K\text{diag}\left(E_{l}\right)K\right)=\frac{1}{M^{2}B}\sum_{b}\left(v_{b}{\left(w\right)}^{T}\text{diag}\left(E_{l}\right)u_{b}\right),\\ {\left(J^{T}J\right)}_{\sigma_{\beta }^{2},\sigma_{\beta }^{2}}=\text{tr}\left(KK\right)=\frac{1}{M^{2}B}\sum_{b}||u_{b}|{|}_{2}^{2},\\ {\left(J^{T}J\right)}_{\sigma_{\beta }^{2},\sigma_{e}^{2}}=\text{tr}\left(K\right)=\frac{1}{M^{2}B}\sum_{b}z_{b}^{T}Wu_{b},\\ {\left(J^{T}J\right)}_{w_{l},\sigma_{e}^{2}}=\text{tr}\left(\text{diag}\left(\eta \right)K\text{diag}\left(E_{l}\right)\right)=\frac{1}{M^{2}B}\sum_{b}z_{b}^{T}Wv_{b}(w),\\ {\left(J^{T}J\right)}_{\sigma_{e}^{2},\sigma_{e}^{2}}=\text{tr}\left(W\right). \end{array}$$

$J{\left(\theta \right)}^{T}\epsilon (\theta )$ is given by

$$\begin{array}{c} {\left(J{\left(\theta \right)}^{T}\epsilon (\theta )\right)}_{\sigma_{\beta }^{2}}=\text{tr}\left(y^{T}W\mathrm{KWy}\right)-J{\left(\theta \right)}^{T}J(\theta )\sigma_{\beta }^{2},\\ {\left(J{\left(\theta \right)}^{T}\epsilon (\theta )\right)}_{w_{l}}=\text{tr}\left(y^{T}W\text{diag}\left(E_{l}\right)K\text{diag}\left(Ew\right)Wy\right)-J{\left(\theta \right)}^{T}J(\theta )w_{l},\\ {\left(J{\left(\theta \right)}^{T}\epsilon (\theta )\right)}_{\sigma_{e}^{2}}=\text{tr}\left(y^{T}Wy\right)-J{\left(\theta \right)}^{T}J(\theta )\sigma_{e}^{2}. \end{array}$$

where

$$\text{tr}\left(y^{T}W\text{diag}\left(E_{l}\right)K\text{diag}\left(Ew\right)Wy\right)=\sum_{m}H_{l,m}$$

Finally the squared error, which we define as S(θ), is given by

$$\begin{array}{c} S(\sigma_{\beta }^{2},w)=||(yy^{T}-\text{Cov}\left(y\right))|{|}_{F}^{2},\\ =\text{tr}\left(\left(yy^{T}-\text{Cov}\left(y\right))(yy^{T}-\text{Cov}\left(y\right)\right)\right),\\ =\text{tr}\left(yy^{T}yy^{T}\right)-2{\left(\begin{array}{c} \sigma_{\beta }^{2}\\ 1\\ \sigma_{e}^{2} \end{array}\right)}^{T}\left(\begin{array}{c} \text{tr}\left(y^{T}Ky\right)\\ \text{tr}\left(y^{T}K_{2}(w)y\right)\\ \text{tr}\left(y^{T}y\right) \end{array}\right)\;\\ +{\left(\begin{array}{c} \sigma_{\beta }^{2}\\ 1\\ \sigma_{e}^{2} \end{array}\right)}^{T}\left(\begin{array}{ccc} \text{tr}\left(KK\right) & \text{tr}\left(KK_{2}(w)\right) & \text{tr}\left(K\right)\\ \text{tr}\left(KK_{2}(w)\right) & \text{tr}\left(K_{2}(w)K_{2}(w)\right) & \text{tr}\left(K_{2}(w)\right)\\ \text{tr}\left(K\right) & \text{tr}\left(K_{2}(w)\right) & N \end{array}\right)\left(\begin{array}{c} \sigma_{\beta }^{2}\\ 1\\ \sigma_{e}^{2} \end{array}\right) \end{array}$$

where

$$\text{tr}\left(K_{2}(w)K_{2}(w)\right)\approx \frac{1}{M^{2}B}\sum_{b}||v_{b}(w)|{|}_{2}^{2}$$

The initial preprocessing step has costs $O(\mathrm{NMLB}+NML^{2})$ in compute and O(NLB) in RAM. The remaining algorithm does not require much RAM in addition to that required in the preprocessing step, so also costs O(NLB) in RAM. Construction of the summary variable $v_{b}(w)=\sum_{l}w_{l}v_{b,l}$ costs O(NLB) in compute. Each iteration of the LM algorithm costs $O(NL^{2}B)$.

It is possible to parallelize GPLEMMA using OpenMPI by partitioning samples across cores, in a similar manner to that used by LEMMA (Kerin and Marchini, 2020). Given that evaluating the objective function $S(\sigma_{\beta }^{2},w)$ is characterized by BLAS level 1 array operations, a distributed algorithm using OpenMPI should have superior runtime versus an the same algorithm using OpenMP as well as providing RAM limited only by the size of a researchers compute cluster.

We perform 10 repeats of the LM algorithm with different initializations, and keep results from the solution with lowest squared error $S(\hat{\theta })$. Each run is initialized with a vector of interaction weights $\tilde{w}$, where each entry set to $\frac{1}{L}$ and a small amount of Gaussian noise is added.

$$\tilde{w}=\frac{1}{L}\vec{1}+N\left(0,\frac{2}{L^{2}}I_{L}\right).$$

To transform the initial weights vector $\tilde{w}$ to the initial parameters *θ*_0_ we let $\left({\hat{\sigma }}_{\beta }^{2},{\hat{\sigma }}_{\gamma }^{2},{\hat{\sigma }}_{e}^{2}\right)$ be solutions to

$$\left({\hat{\sigma }}_{\beta }^{2},{\hat{\sigma }}_{\gamma }^{2},{\hat{\sigma }}_{e}^{2}\right)=\min_{\sigma_{\beta }^{2},\sigma_{\gamma }^{2},\sigma_{e}^{2}}||yy^{T}-\left(\sigma_{\beta }^{2}K+\sigma_{\gamma }^{2}K_{2}(\tilde{w})+\sigma_{e}^{2}I\right)|{|}_{F}^{2}.$$

The GPLEMMA algorithm is then initialized with $\theta_{0}=\left({\hat{\sigma }}_{\beta }^{2},w,{\hat{\sigma }}_{e}^{2}\right)$ where $w=\sigma_{\gamma }\tilde{w}$.

#### 2.3.4 LEMMA

The LEMMA model is given by

(12) y=Cα+Xβ+(η⊙X)γ+ϵ,

 

(13) $$\beta \sim \psi N\left(0,\frac{\sigma_{\beta 1}^{2}}{M}I_{M}\right)+(1-\psi )N\left(0,\frac{\sigma_{\beta 2}^{2}}{M}I_{M}\right),$$

 

(14) $$\gamma \sim \pi N\left(0,\frac{\sigma_{\gamma 1}^{2}}{M}I_{M}\right)+(1-\pi )N\left(0,\frac{\sigma_{\gamma 2}^{2}}{M}I_{M}\right),$$

where η=Ew is the linear environmental score (ES). The use of the MoG priors makes it harder to analytically integrate out the parameters. The LEMMA algorithm uses a Variational Bayes approach to first estimate the ES and fit the whole genome regression pararmeters *β* and *γ*. The primary use of the LEMMA model is for testing individual SNPs for GxE effects. However, LEMMA can also estimate GxE heritability. It uses the VB algorithm to estimate the ES, and then plugs this estimate into Eq 6 and uses a randomized Haseman–Elston linear regression to estimate the variance components.

#### 2.3.5 Relationship between MEMMA and GPLEMMA

Comparing Equation (3) with Section 6, suggests that the GPLEMMA model can be expressed at the MEMMA model with the added constraint that

$$\Lambda =w\gamma^{T}$$

where $\Lambda ={\left[\lambda_{1},\ldots ,\lambda_{L}\right]}^{T}$ is the *L *×* M* matrix of GxE effect sizes in MEMMA for the *L* environments and *M* SNPs.

We can expect the two models to give similar heritability estimates, under the simplifying assumptions that GxE interactions do occur with a single linear combination of the environments and that the set of random variables $\left\{g,{\left(E_{l}⊙g\right)}_{l=1}^{L}\right\}$ is mutually independent. Let g∼N(0,K) and $\epsilon ∼N(0,\sigma_{e}^{2}I)$. Then connection between the two models is revealed by observing

$$\begin{array}{c} y=N\left(C\alpha ,\sigma_{\beta }^{2}K+\sigma_{\gamma }^{2}K_{2}(w)+\sigma_{e}^{2}I\right),\\ =\sigma_{\beta }g+\left(\sigma_{\gamma }\sum_{l}w_{l}E_{l}\right)⊙g+\epsilon ,\\ =\sigma_{\beta }g+\sum_{l}\sigma_{w_{l}}E_{l}⊙g+\epsilon ,\\ =N\left(0,\sigma_{\beta }^{2}K+\sum_{l}\sigma_{w_{l}}^{2}E_{l}⊙K⊙E_{l}^{T}+\sigma_{e}^{2}I\right), \end{array}$$

where $\sigma_{w_{l}}^{2}=\sigma_{\gamma }^{2}w_{l}^{2}$. Thus we should expect both models to have the same estimate for the proportion of variance explained by GxE interaction effects.

Even in the case that MEMMA and GPLEMMA have the same expected heritability estimate, there are still some differences between the two. GPLEMMA is a constrained model, so the variance of its heritabiity estimates may be smaller. Further, although ${\hat{\sigma }}_{w_{l}}^{2}$ is proportional to the square of the weights used to construct the ES the sign of the interaction weight *w_l_* has been lost. Thus it is not possible to reconstruct an ES for use in single SNP testing using MEMMA.

### 2.4 Simulated data

We carried out simulation studies to compare the performance of MEMMA, LEMMA and GPLEMMA in a variety of different scenarios, using three different phenotype simulation strategies; a baseline phenotype that was simulated according to the LEMMA model (Kerin and Marchini, 2020), a phenotype which generalizes the LEMMA model to three orthogonal ESs and a misspecified phenotype that had squared dependancy on a heritable environmental variable *S*.

The simulations use real data subsampled from genotyped SNPs in the UK Biobank (Bycroft *et al.*, 2018), drawing SNPs from all 22 chromosomes in proportion to chromosome length and using unrelated samples of mixed ancestry (N=25k; 12 500 white British, 7500 Irish and 5000 white European, N=50k; 29 567 white British, 7500 Irish and 12 568 white European, N=100k; 79 567 white British, 7500 Irish and 12 568 white European; using self-reported ancestry in field *f.21000.0.0*). All samples were genotyped using the UKBB genotype chip and were included in the internal principal component analysis performed by the UK Biobank. Environmental variables were simulated from a standard Gaussian distribution.

Let *N* be the number of individuals, *M* the total number of SNPs, *L* the total number of environmental variables and $h_{G}^{2}$ and $h_{\text{GxE}}^{2}$ the herirtability of main effects and GxE effects. The baseline phenotype was simulated using the model

$$\begin{array}{c} y=C\alpha +X\beta +(\eta ⊙X)\gamma +\epsilon ,\\ \eta =Ew,\\ \epsilon ∼N(0,I), \end{array}$$

where *X* represents the *N *×* M* genotype matrix after columns have been standardized to have mean zero and variance one, *C* is the first principle component of the genotype matrix and *E* is the *N *×* L* matrix of environmental variables. In all simulations *α* was set such that Cα explained one percent of trait variance. The interaction weight vector *w* contained $L^{\text{active}}$ non-zero elements, which were drawn from a decreasing sequence

$$w_{i}=\left\{ \begin{array}{cc} {\left(-1\right)}^{i}\left(1-\frac{i}{2L^{\text{active}}}\right) & i\leq L^{\text{active}},\\ 0 & o/w. \end{array}\right.$$

The effect size parameters *β* and *γ* were simulated from a spike and slab prior such that the number of non-zero elements was governed by $M_{G}$ and $M_{\text{GxE}}$ for main and interaction effects respectively. Non-zero elements were drawn from a standard Gaussian, and then subsequently rescaled to ensure that the heritability given by main and interaction effects was $h_{g}^{2}$ and $h_{\text{GxE}}^{2}$ respectively. We chose a set of default parameters: $N=25K;M=100K;L=30;L^{\text{active}}=6,M_{G}=2500;M_{\text{GxE}}=1250;h_{g}^{2}=20\%;h_{\text{GxE}}^{2}=5\%$, and then varied one parameter at a time to examine the effects of sample size, number of environments, number of non-zero SNP effects and GxE heritability. In addition, we investigated performance using a larger baseline simulation with N=100K samples and M=300K variants. The first genetic principal component was provided as a covariate to all methods.

**Fig. 1.**
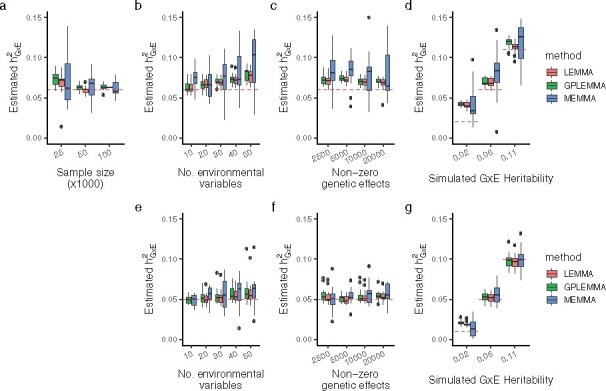
PVE estimation. Estimates of the proportion of variance explained by GxE effects by LEMMA, MEMMA and GPLEMMA whilst varying the number of environments, the number of active environments, the number of non-zero SNP effects and GxE heritability. All simulations constructed with the baseline phenotype. **a–d** Simulations results using N=25K samples and M=100K variants by default, **e–g** show simulation results using N=100K samples and M=300K variants. Results from 15 repeats shown

Figure 1 compares estimates of the proportion of variance explained (PVE) by GxE effects from all three methods. In general, all methods had upwards bias that decreased with sample size and increased with the number of environments. While heritability estimates from LEMMA and GPLEMMA appeared quite similar, estimates from MEMMA had much higher variance and also appeared to have higher upwards bias as the total number of environments increase. All the methods exhibited less bias in the larger simulations with N=100K samples and M=300K variants *(*Fig. 1e–g). Supplementary Figure S1 shows the estimated PVE by main effects from the same set of simulations. In general the estimated PVE by main effects from the three methods was extremely similar.

**Fig. 2.**
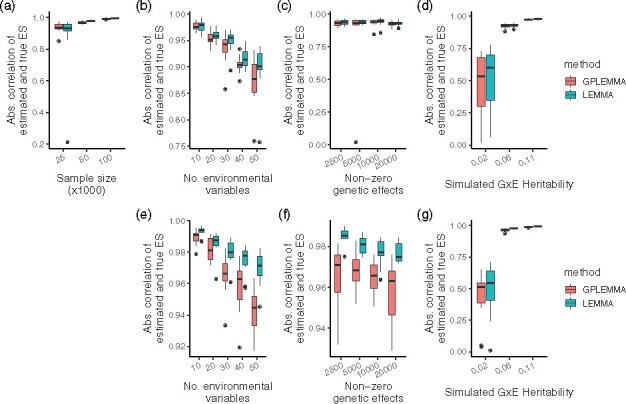
Correlation between the true ES and the inferred ES. Absolute correlation between the true ES and the ES inferred by LEMMA and GPLEMMA whilst varying the number of environments, the number of active environments, the number of non-zero SNP effects and GxE heritability. All simulations constructed with the baseline phenotype. **a–d** Simulations results using N=25K samples and M=100K variants by default, **e–g** simulation results using N=100K samples and M=300K variants. Results from 15 repeats shown

Figure 2 compares the absolute correlation between the simulated ES and the ES inferred by LEMMA and GPLEMMA (note that MEMMA does not provide an estimate of the ES). In general, the estimated ES from GPLEMMA had slightly lower absolute correlation with the true ES than the estimated ES from LEMMA. LEMMA models SNP effects directly, so can estimate the SNPs involved in the interaction, and this is likely the reason for the small improvement in the accuracy of the ES. In large sample sizes (N=100k with M=300k SNPs), both methods achieve a correlation of over 0.98 with the simulated ES.

Next, we tested the methods against a generalized phenotype with three orthogonal ESs that interacted with disjoint sets of SNPs. More explicitly, the generalized phenotype was simulated from the following model

$$y\sim N\left(C\alpha +X\beta +\sum_{k=1}^{3}\eta_{k}⊙X\gamma_{k},I_{N}\right),$$

where $\eta_{k}=EW_{k}$ is an *N*-vector and *W* is an *L *×* K* matrix of environmental weights with *K *=* *3. *W* contained $L^{\text{active}}=6$ rows with non-zero elements drawn from a standard Gaussian distribution, and columns were pairwise orthogonal. Similarly, *γ_k_* denotes an *M*-vector where the non-zero elements of *γ_k_* and *γ_j_* are disjoint. SNP coefficients were drawn from a spike and slab prior in a similar manner to the baseline phenotype with $M_{G}=2500$ and $M_{\text{GxE}}=1250$.

**Fig. 3.**
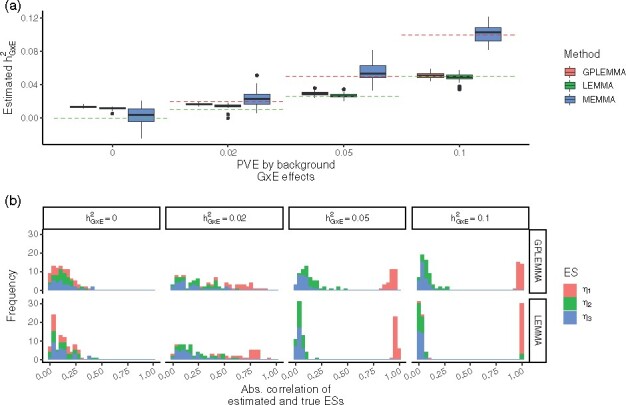
Comparison with 3 simulated ESs. **a** Estimates of the proportion of variance explained by GxE effects by LEMMA, MEMMA and GPLEMMA whilst varying the simulated GxE heritability. The red dashed line indicates the total GxE heritability. The dashed green line indicates the heritability of the first GxE component *η*_1_. **b** The (absolute) correlation between the estimated ES and the three simulated ESs. Simulations constructed using N=100K samples and M=100K variants. Results from 15 repeats shown

Figure 3 displays results from a set of simulations with N=100k samples and M=100k SNPs. The three ESs were scaled such that the singular values of Λ where decreasing (with values 80, 60, 40). Figure 3b shows that the ES estimated by both LEMMA and GPLEMMA was, in general, highly correlated with the ES with highest singular value when the simulated GxE heritability was over 0.05. In this scenario, estimates of GxE heritability from both methods was centered around the true simulated heritability of that ES (see Fig. 3a). However at lower levels of simulated GxE heritability, both methods generally failed to identify one of the true ESs (consistent with Fig. 2). In contrast, the estimates of GxE heritability from MEMMA were centered on the sum of the GxE heritabilities from all three ESs. This suggests that running both GPLEMMA and MEMMA may help to elucidate the architecture of GxE interactions for a given trait.

For a more challenging scenario, we reran the high GxE heritability ($h_{\text{GxE}}^{2}=0.1$) simulation using a larger number of SNPs (M=300K) so that *N *>* M* and scaled the ESs such that the singular values corresponding to the first two ESs were roughly equal (the singular values were 80, 78, 60). In this scenario the ES estimated by GPLEMMA did not correlate well with any one of the three simulated ESs, but was highly correlated with a linear combination of them (Supplementary Fig. S2). We hypothesize that with a large number of ‘null’ variants GPLEMMA becomes less able to identify a single ES and instead infers a mixture. In contrast, LEMMA consistently identified one of the simulated ESs. The GxE heritability estimates from all three methods were roughly as before, but with some inflation (Supplementary Fig. S3).

In the third batch of simulation results we used a misspecified phenotype, with squared dependancy on a heritable environmmental variable *S*. The misspecified phenotype was simulated using the model

$$y_{\text{misspec}}=\alpha_{s}S^{2}+y_{\text{baseline}},$$

where *S* was simulated to have heritability of 30% with 2500 causal SNPs drawn from a spike and slab prior, and a range of values for *α_s_* was used to vary the strength of the non-linear relationship between *y* and *S*.

Supplementary Figure S4 compares MEMMA, GPLEMMA and LEMMA in a simulation where the functional form of a heritable environmental variable was misspecified (or more specifically; the phenotype depended on the squared effect of a heritable environment). All methods were first tested without any attempt to control for model misspecification, and second using a preprocessing strategy where each environment was tested independently for squared effects on the phenotype and any squared effects with p-value <0.01/L were included as covariates. These are referred to as *(−SQE)* and *(+SQE)* respectively in the figures. Using the *(−SQE)* strategy, all methods showed upwards bias in estimates of GxE heritability that increased with the strength of the squared effect on the phenotype (Supplementary Fig. S4b). Model misspecification also caused bias in the ES of both GPLEMMA and LEMMA, however bias in the ES from GPLEMMA appeared to be much worse (Supplementary Fig. S4a). Using the (+SQE) strategy, all GxE heritability estimates were unbiased, consistent with earlier simulation results.

**Fig. 4.**
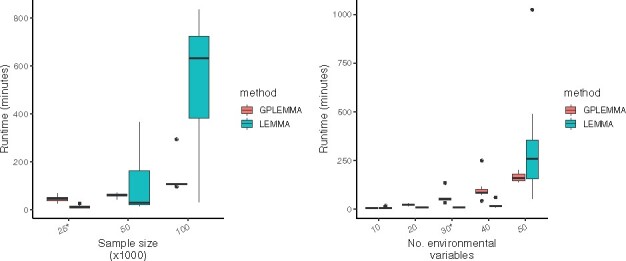
Computational cost of GPLEMMA. Comparison of the computational cost of LEMMA and GPLEMMA as (**a**) sample size and (**b**) the number of environments increases. Runtime shown on a $\;{\text{log}}_{10}$ scale. By default each run used four cores, N=25k samples, *L *=* *30 environments and 10 random starts of the Levenburg-Marquardt algorithm. Results from 10 repeats shown. Runtime for LEMMA excludes time spent on single SNP hypothesis testing

Figure 4 shows a comparison of the runtime of LEMMA and GPLEMMA as the sample size and number of environments varied. The figure shows that on relatively small datasets (N=25k samples or *L *=* *10 environmental variables) LEMMA was slightly faster, however on large datasets GPLEMMA is faster due to superior algorithmic complexity. To give a direct comparison, on a simulated data with N=100k samples, M=100k SNPs and *L *=* *30 environmental variables using 4 cores for each run, LEMMA took an average of 648 minutes to run whereas GPLEMMA took an average of 233 minutes.

Supplementary Figure S5 displays simulation results on the computational complexity of GPLEMMA. Supplementary Figure S5a and b shows that GPLEMMA achieved perfect strong scaling (A parallel algorithm has perfect strong scaling if the runtime on *T* processors is linear in $\frac{1}{T}$, including communication costs.) on the range of cores tested. This suggests that GPLEMMA has superior scalability to LEMMA, as for LEMMA the speedup due to increased cores began to decay after the number of samples per core dropped below 3000 (Kerin and Marchini, 2020).

Time to compute the preprocessing step and solve the non-linear least squared problem are shown in Supplementary Figure S5c–f, while the number of environments and sample size were varied. As expected, the preprocessing step appeared to be linear in both the number of environments and sample size. Time to solve the non-linear least squares problem appeared to be quadratic in the number of environments and approximately linear in sample size *N*. As a single LM iteration should have complexity $O(NL^{2}B)$, this suggests that the number of iterations required for convergence of the LM algorithm was independent of sample size and the number of environments (at least for the range of values tested).

Finally, we tested GPLEMMA in simulation where we simulated ordinal environmental variables, created using a binomial distribution *Bin*(*n*, *p*) where *n* with n∈{3,4,5} levels and p∼U(0,0.5). Comparing GPLEMMAs performance in these simulations with previous results using a continuous environmental variable (Supplementary Fig. S6) suggests that GPLEMMA is not sensitive to the choice of ordinal or continuous environmental variables.

### 2.5 Analysis of UK Biobank data

To compare GPLEMMA and LEMMA on real data we ran both methods on body mass index (log BMI), systolic blood pressure (SBP), diastolic blood pressure (DBP) and pulse pressure (PP) measured on individuals from the UK Biobank. We filtered the SNP genotype data based on minor allele frequency (≥0.01) and IMPUTE info score (≥0.3), leaving approximately 642 000 variants per trait. We used 42 environmental variables from the UK Biobank, similar to those used in previous GxE analyses of BMI in the UK Biobank (Moore *et al.*, 2019; Young *et al.*, 2016). After filtering on ancestry and relatedness, sub-setting down to individuals who had complete data across the phenotype, covariates and environmental factors we were left with approximately 280,000 samples per trait. The sample, SNP and covariate processing and filtering applied is the same as that reported in the LEMMA paper (Kerin and Marchini, 2020).

Table 1 shows the estimates and standard errors for SNP main effects ($h_{G}^{2}$) and GxE effects ($h_{\text{GxE}}^{2}$) for GPLEMMA and LEMMA applied to the 4 traits. In all cases there is good agreements between the estimates from both methods.

**Table 1.** Comparison of GPLEMMA and LEMMA on 4 UK Biobank traits

Trait	$h_{G}^{2}$ (s.e)		$h_{\text{GxE}}^{2}$ (s.e)	
	GPLEMMA	LEMMA	GPLEMMA	LEMMA
log BMI	0.256 (0.078)	0.259 (0.069)	0.074 (0.008)	0.071 (0.009)
PP	0.230 (0.042)	0.233 (0.039)	0.063 (0.007)	0.075 (0.018)
SBP	0.237 (0.057)	0.240 (0.053)	0.036 (0.003)	0.033 (0.003)
DBP	0.273 (0.037)	0.277 (0.034)	0.021 (0.003)	0.014 (0.001)

*Note*: Heritability estimates obtained using genotyped SNPs.

Finally we ran RHE-regression on the four UK Biobank traits whilst controlling for the same set of covariates. Heritability estimates from RHE-regression were marginally higher than those obtained by LEMMA and GPLEMMA (see Supplementary Table S1).

## 3 Discussion

Primarily this article develops a novel randomized Haseman–Elston non-linear regression approach for modelling GxE interactions of quantitative traits in large genetic studies with multiple environmental variables. This approach estimates GxE heritability at the same time as estimating the linear combination of environmental variables (called an ES) that underly that heritability. This general idea was pioneered in our previous approach LEMMA (Kerin and Marchini, 2020) which used a whole-genome regression approach to learn the ES, and this was then used in a randomized Haseman–Elston approach to estimate GxE heritability. The GPLEMMA approach introduced in this article does not need that first whole-genome regression step, and this leads to substantial computational savings. The model underlying GPLEMMA is very similar to that in LEMMA, but implicity assumes a Gaussian distribution for main SNP effects and GxE effects at each SNP.

We compared GPLEMMA to a simpler approach, which we called MEMMA, that estimates GxE heritability of each environmental variable in a joint model, but does not attempt to find the best linear combination of them. We found that estimates of GxE heritability from MEMMA had higher variance than estimates from LEMMA and GPLEMMA, suggesting that the usefulness of MEMMA might be limited. Results from LEMMA and GPLEMMA were very similar, both in terms of estimating the ES and GxE heritability. The primary advantage of GPLEMMA over LEMMA is in computational complexity, as the empirical complexity of GPLEMMA appeared to be linear in sample size whereas LEMMA was shown to be super-linear (Kerin and Marchini, 2020).

The methods LEMMA, GPLEMMA and MEMMA have all been developed for quantitative traits, and we have not explored their use when applied directly to binary traits, as if they were continuous, as was carried out in Pazokitoroudi *et al.* (2020). Developing GPLEMMA to directly model binary traits is a direction for future work. It maybe that transformations that exist for single component LMMs to convert heritability estimates to the liability scale may be also work here.

In the future it may also be interesting to extend the GPLEMMA model to partition variance using multiple orthogonal linear combinations of environmental variables. This could be expressed using the model

(15) $$y=C\alpha +X\beta +\sum_{j=1}^{J}\left(\eta_{j}⊙X\right)\gamma_{j}+\epsilon ,$$

where $\eta_{j}=Ew_{j}$ is an N-vector, *w_j_* is an *L*-vector and $w_{j}⊥w_{k}\forall j,k\in \{1,\ldots ,J\}$.

LEMMA is also able to perform single SNP hypothesis testing whereas GPLEMMA (currently) does not. The linear weighting parameter *w* from GPLEMMA could be used to initialize LEMMA, or the estimated ES could be used as a single environmental variable in LEMMA. Exploring these, and other, approaches is future work.

While we are enthusiastic about the potential of GPLEMMA (and LEMMA) to elucidate the contribution of GxE interactions to disease traits, we suggest that more care is needed than a standard genetic heritability analysis for a number of reasons. As our simulations show, including a variable as an ’environment’ that is itself under genetic control can lead to bias if the relationship between that variable and trait of interest is not well modelled, and this should be carefully considered. Also, GPLEMMA is only able to assess the contribution of those variables included in the model. It may well be the case that a relevant environment is not available and so its GxE contribution cannot be assessed. Finally, the scale the measured trait can impact results (Kerin and Marchini, 2020) so it can be useful to assess results on a range of scales.

## Supplementary Material

btaa1079_Supplementary_Data
