# Supplementary material for: A non-linear regression method for estimation of gene–environment heritability
Source: Bioinformatics. 2020 Dec 26;36(24):5632–9. doi: 10.1093/bioinformatics/btaa1079 (PMC8023682; doi:10.1093/bioinformatics/btaa1079)
Supplement: btaa1079_Supplementary_Data [file btaa1079_supplementary_data.pdf]

Supplementary Material for 'A non-linear regression method for estimation of gene-environment heritability'

Matthew Kerin[1] & Jonathan Marchini[2]

[1] *Wellcome Trust Center for Human Genetics, Oxford, OX3 7BN, UK.*

[2] *Regeneron Genetics Center, Tarrytown, NY 10591, USA.*

Corresponding author *Jonathan Marchini (jonathan.marchini@regeneron.com)*

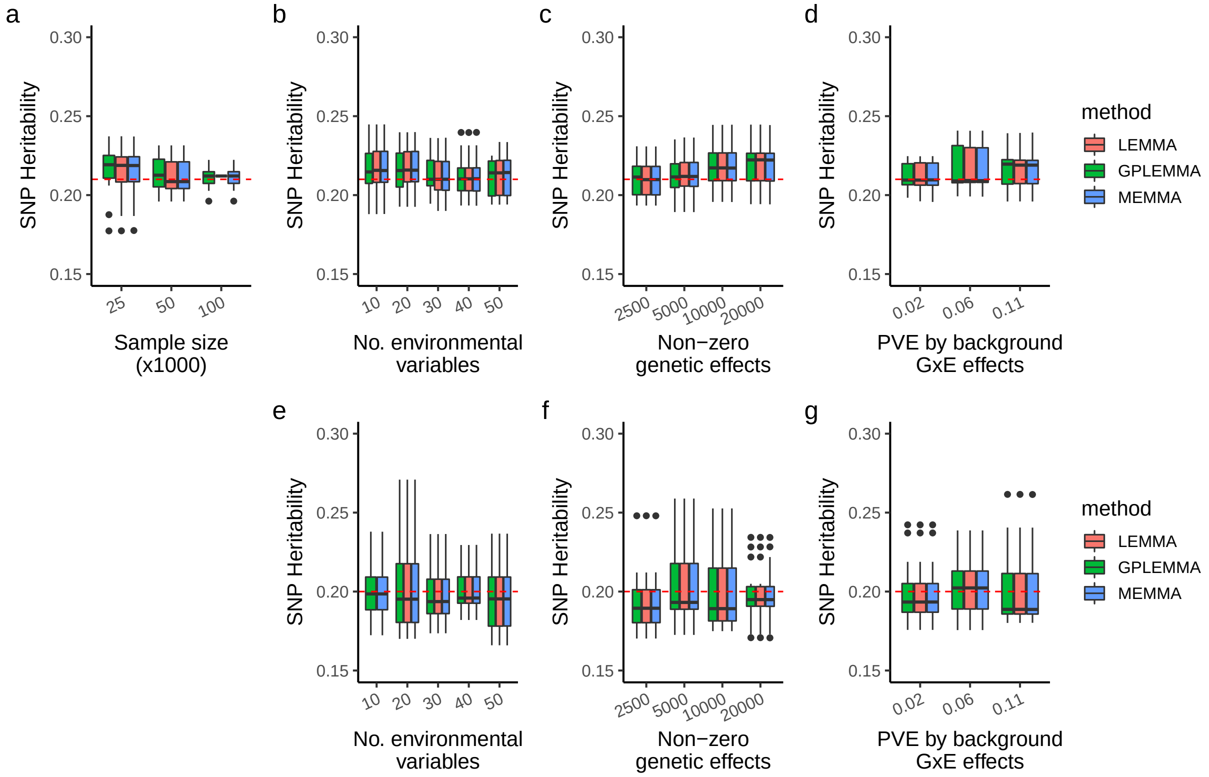

Figure S1: **PVE estimation**. Estimates of the proportion of variance explained by main effects by LEMMA, MEMMA and GPLEMMA on baseline simulations with using $N = 25K$ samples and $M = 100K$ variants, whilst varying sample size (a), the number of environments (b), the number of non-zero SNP effects (c) and GxE heritability (d). Panels (e-g) shows results of simulations with $N = 100K$ samples and $M = 300K$ variants, whilst varying the number of environments (e), the number of non-zero SNP effects (f) and GxE heritability (g).

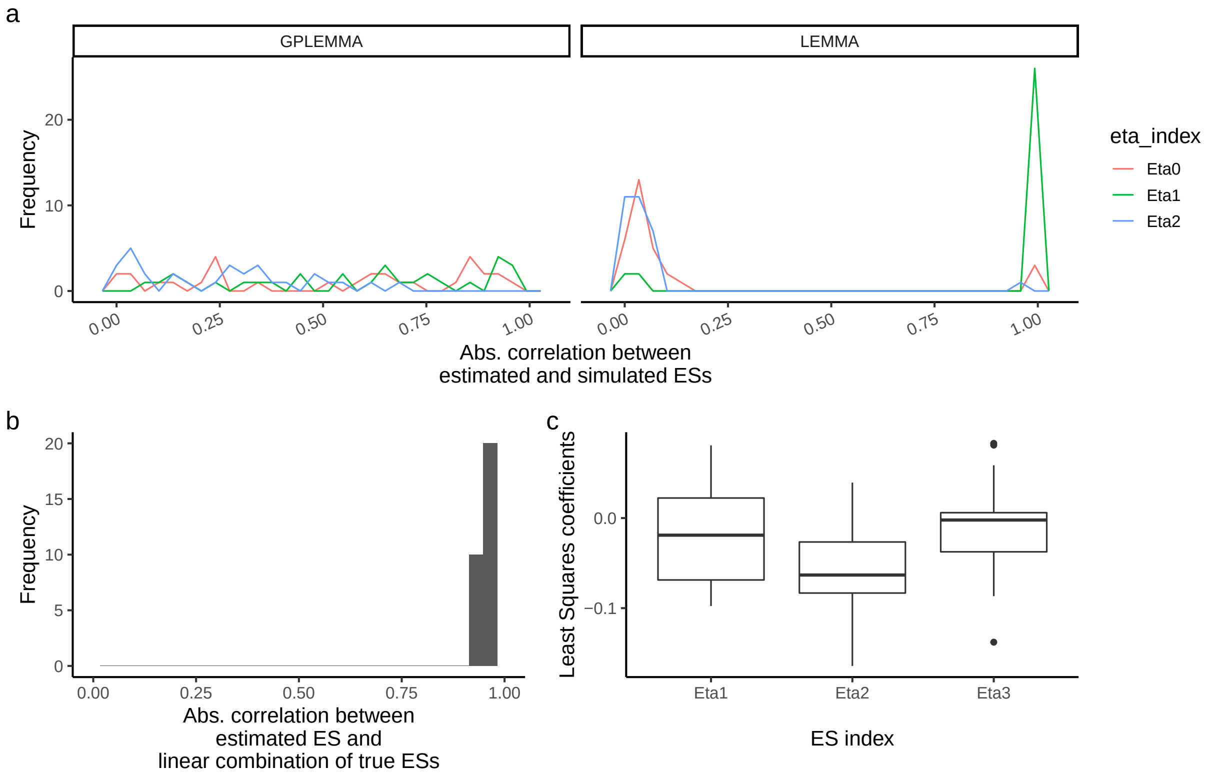

Figure S2: **Results of methods from more challenging scenario with 3 ESs.** Panel (a) shows the (absolute) correlation between the estimated ES and the three simulated ESs. For panel (b) we use ordinary least squares to fit a linear model of the simulated ESs against the estimated ES, and display correlation of the fitted vector with the estimatd ES. Panel (c) shows the coefficients from this linear model. Simulations constructed using $N = 100K$ samples and $M = 300K$ variants. Results from 15 repeats shown.

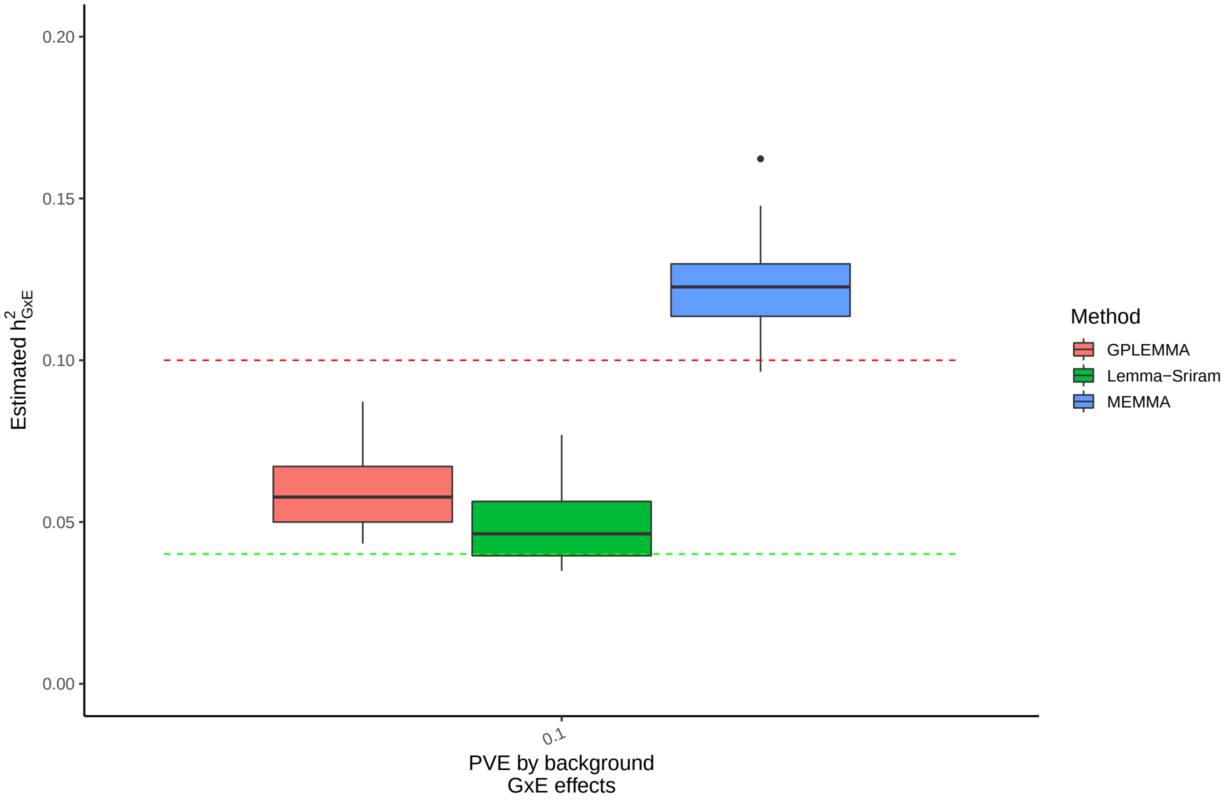

Figure S3: **PVE estimates in more challenging scenario with 3 ESs.** Estimates of the proportion of variance explained by GxE effects by LEMMA, MEMMA and GPLEMMA. The red dashed line indicates the total GxE heritability. The dashed green line indicates the heritability of the first GxE component η_1.

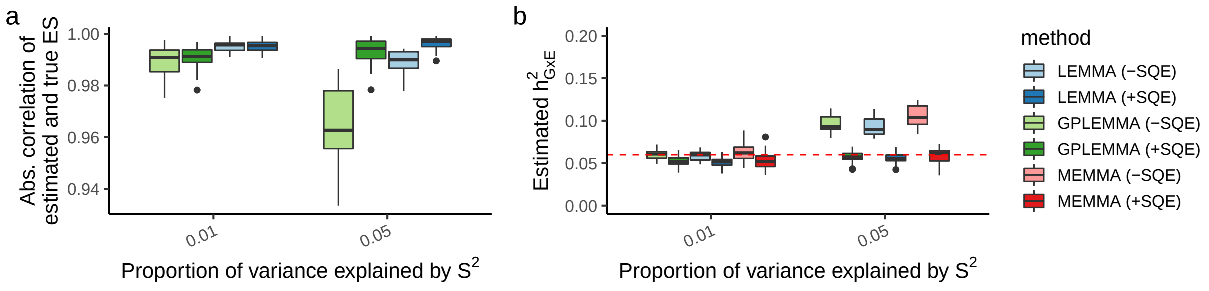

Figure S4: **Comparison on simulations with a misspecified heritable environment** Estimated proportion of trait variance explained by GxE effects is shown on the left, absolute correlation between the inferred ES and the true ES shown in the right. Results shown using LEMMA, MEMMA and GPLEMMA. Phenotypes simulated with a squared effect from a heritable confounder. Results from 20 repeats shown. Abbreviations; (-SQE), no attempt to control for squared effects; (+SQE), squared effects with $p < 0.01$ (Bonferroni correction for multiple envs) included as covariates

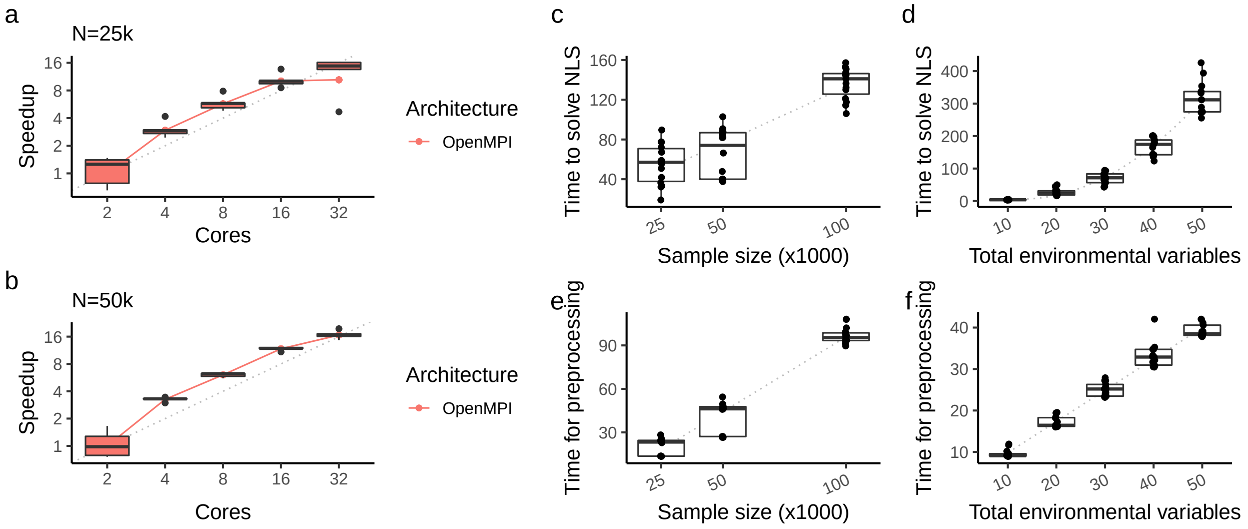

Figure S5: **Computational complexity of GPLEMMA in simulation**. Strong scaling of GPLEMMA using OpenMPI to parrallelise across cores with (a) $N = 25k$ samples and (b) $N = 50k$ samples. Comparison of the runtime of the Levenburg-Marquardt non-linear least squares (NLS) algorithm (c, d) and runtime of the preprocessing step (e, f). By default each run used; four cores, $N = 25k$ samples, $L = 30$ environments and 10 random starts of the Levenburg-Marquardt algorithm. Results from 15 repeats shown.

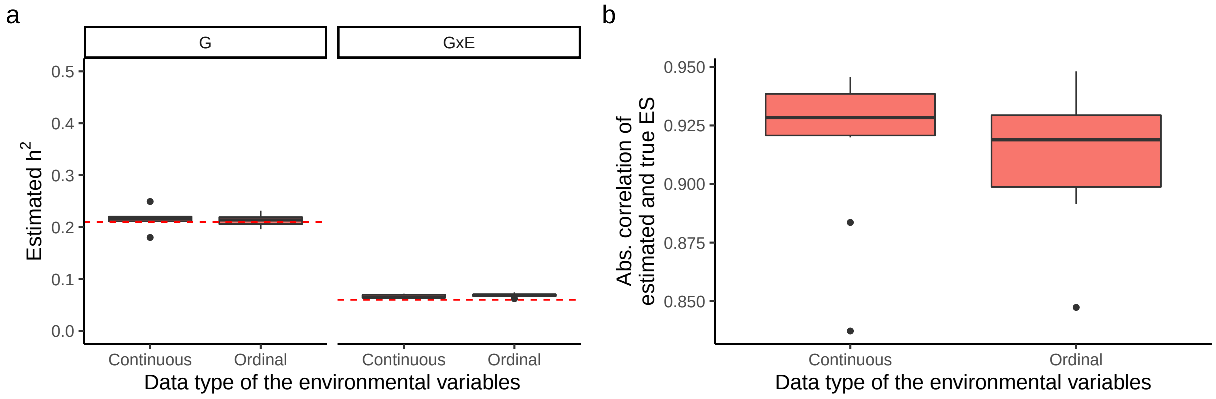

Figure S6: **Comparison of GPLEMMA on continuous and ordinal environmental data.** In the continuous simulation, environmental data was simulated from a standard gaussian distribution. In the ordinal simulation, environmental data was simulated from a binomial distribution $Bin(n, p)$ where n was between $3-5$ and $p \sim U(0, 0.5)$. Each run used four cores, $N = 25k$ samples, $M = 100k$ SNPs, $L = 30$ environments (of which 6 were active) and 10 random starts of the Levenburg-Marquardt algorithm. The dotted red line indicates the true PVE by main and interaction effects. Results from 10 repeats shown.

Trait	h^2_{G} (s.e)		
	RHE	GPLEMMA	LEMMA
log BMI	0.263 (0.069)	0.256 (0.078)	0.259 (0.069)
PP	0.234 (0.039)	0.230 (0.042)	0.233 (0.039)
SBP	0.241 (0.052)	0.237 (0.057)	0.240 (0.053)
DBP	0.279 (0.033)	0.273 (0.037)	0.277 (0.034)

Table S1: **Estimates of the proportion of variance explained by SNP additive effects on 4 UK Biobank traits**. We compared PVE estimates from RHE-regression, LEMMA and GPLEMMA. All methods ran on genotyped SNPs, and controlled for the same covariates. LEMMA and GPLEMMA also modelled the proportion of variance explained by GxE effects.
